# Esophageal Cancer Associated Immune Genes as Biomarkers for Predicting Outcome in Upper Gastrointestinal Tumors

**DOI:** 10.3389/fgene.2021.707299

**Published:** 2021-07-19

**Authors:** Chuanhui Zhu, Qianqian Xia, Bin Gu, Mengjing Cui, Xing Zhang, Wenjing Yan, Dan Meng, Siyuan Shen, Shuqian Xie, Xueliang Li, Hua Jin, Shizhi Wang

**Affiliations:** ^1^Department of Gastroenterology, The First Affiliated Hospital, Nanjing Medical University, Nanjing, China; ^2^Department of Gastroenterology, Nanjing BenQ Medical Center, The Affiliated BenQ Hospital, Nanjing Medical University, Nanjing, China; ^3^Key Laboratory of Environmental Medicine Engineering, Ministry of Education, School of Public Health, Southeast University, Nanjing, China; ^4^Department of Neurosurgery, Zhongda Hospital, School of Medicine, Southeast University, Nanjing, China; ^5^Clinical Laboratory, Affiliated Tumor Hospital of Nantong University (Nantong Tumor Hospital), Nantong, China

**Keywords:** esophageal cancer, prognostic biomarker, head and neck cancers, gastric cancer, the upper gastrointestinal tumors

## Abstract

Esophageal cancer (EC) is the seventh most common tumor in the world, ranking the sixth leading cause of cancer death, with a 5-year survival rate of 15-25%. Therefore, reliable prognostic biomarkers are needed to effectively predict the prognosis of EC. In this study, the gene profile information of the EC cohort served as a training set, which was derived from TCGA and Immport databases. GO and KEGG enrichment analysis was performed on the differential genes in normal and tumor groups of EC. The immune genes in differentially expressed genes (DEGs) were further obtained for univariate and multivariate Cox and Lasso regression analysis, and 6 independent immune genes (*S100A3*, *STC2*, *HSPA6*, *CCL25*, *GPER1*, and *OSM*) associated with prognosis were obtained to establish an immune risk score signature (IRSS). The signature was validated using head and neck cancers (HNSC) and gastric cancer (GC)in upper gastrointestinal malignancies as validation sets. The Kaplan-Meier results showed that the prognosis of the high-risk group was significantly favorable than that of the low-risk group in both the training set (*P* < 0.001; HR = 3.68, 95% CI = 2.14−6.35) and the validation set (*P* = 0.010; HR = 1.43, 95% CI = 1.09−1.88). A nomogram combining multiple clinical information and IRSS was more effective than a single independent prognostic factor in predicting outcome. This study explored the potential link between immunity and EC, and established and validated prognostic biomarkers that can effectively predict the prognosis of EC, HNSC and GC based on six immune genes.

## Introduction

Esophageal cancer (EC) is the 7th most common tumor in the world (Global Burden of Disease Cancer Collaboration et al., 2018), ranking the 6th leading cause of cancer death, which seriously threatens human health (Bray et al., 2018). According to the data, it is estimated that 456,000 new cases of EC were reported worldwide in 2012, half of which were in China (Zhu et al., 2016). EC mainly includes two histological subtypes, esophageal squamous cell carcinoma (ESCC) and esophageal adenocarcinoma (EA), accounting for more than 95% of esophageal malignancies, of which ESCC is more common (Enzinger and Mayer, 2003). Smoking, alcohol consumption, chronic gastroesophageal reflux disease, obesity are critical risk factors for the occurrence of the disease (Huang and Yu, 2018). Unfortunately, most patients are already at an advanced stage at diagnosis, therefore the curative ratio is low and the prognosis is poor (Ferlay et al., 2015). In recent years, despite the application of new diagnostic and therapeutic techniques that have improved the survival rate of EC patients (Vendrely et al., 2018), the 5-year overall survival (OS) rate is still unsatisfactory, fluctuating between 15 and 25% (Short et al., 2017). Therefore, it is urgent to find robust biomarkers to predict the prognosis of EC patients and provide potential therapeutic targets.

Inflammation has been well known to be a complex biological response in which the human immune system attempts to eliminate the stimulus of inflammation and initiate repair and regeneration (Wallach et al., 2014; Karin and Clevers, 2016). Inflammatory response plays a pivotal role in tumorigenesis, development and metastasis (Taniguchi and Karin, 2018). For instance, the expression of immune-related genes such as interleukin (IL)-6 members, including IL-11, IL-27, IL-31, leukemia inhibitory factor, and oncostatin M (*OSM*), affect tumor cell proliferation, survival, inflammation, and metabolism (Taniguchi and Karin, 2014). The occurrence of EC is closely correlated to inflammation. It is well known that EA is inflammation-related cancer (O’Sullivan et al., 2014). Chronic inflammation has also been proved to be a crucial factor in the development of ESCC. On the one hand, oxidative and genotoxic stresses caused by smoking, drinking and carcinogens trigger inflammation, on the other hand, oral microbiota disorders, human papillomavirus (HPV) infection, and improper diet can also cause inflammation. EC cells can inhibit the body’s anti-tumor immunity through inflammation-related mechanisms such as immune checkpoints, secretory factors and negatively regulated immune cells (Diakowska and Krzystek-Korpacka, 2020).

Since immune inflammation is a vital process in triggering tumorigenesis, identifying whether immunity affects the prognosis of patients remains an active area of research. Several studies have reported that tumor prognosis-related models have been established to predict patient survival (Huang et al., 2019; Shen et al., 2019; Qu et al., 2020). However, there are few studies on the establishment of prognostic models for EC, let alone immune-related ones. In the present study, we used the Cancer Genome Atlas (TCGA) database to explore the correlation between immune mechanisms and the occurrence of EC and established a novel risk score signature based on immune genes to effectively predict the outcome of EC patients as well as provide a potential clinical combination therapy. Taken together, our findings highlight the functional role of immune-related signatures and reveal potential prognostic biomarkers for ECs to predict the prognosis of upper gastrointestinal tumors.

## Materials and Methods

### Data Collection and Processing

The datasets of esophageal cancer (TCGA-ESCA) and head and neck cancer (TCGA-HNSC), including their gene expression profiles, clinic information and survival information, were downloaded from the UCSC database^1^. EC samples with prognostic information were collected as a training set, consisting of 162 tumor samples and 11 normal samples. And a total of 500 patients with HNSC containing prognostic information were collected as a validation set. Patients with an OS of fewer than 60 days were removed because their cause of death may not be attributable to tumors.

From the Gene List module of the Immunology Database and Analysis Portal (ImmPort) database^2^, we downloaded complete gene names directly, totaling 2483 immune-related genes (Supplementary Table 1).

### Differential Expression Analysis

Based on the expression of genes in EC, we first performed a differential expression analysis to identify genes differentially expressed in normal and tumor groups. Briefly, differentially expressed genes (DEGs) were obtained using the “limma” software package in R. Among them, Log_2_| FC| > 1 and false discovery rate (FDR) < 0.25 were the criteria. “ggplot2,” “Cairo,” and “ggrepel” packages in the R were used to plot volcanoes to visualize the DEGs.

### Gene Ontology (GO) and Kyoto Encyclopedia of Genes and Genomes (KEGG) Analysis

To identify potential biological processes and enrichment pathways of DEGs, GO, and KEGG was performed using the cluster Profilter R package. KEGG is a type of gene annotation, a database that integrates genomic, chemical and systematic functional information. Go database mainly describes gene characteristics in different dimensions and levels, involving cell composition, biological process and molecular function. The adjusted *P*-value less than 0.05 was considered statistically significant.

### Establishment of Immune Risk Scoring Signature (IRSS) for Prognosis

A total of 1734 immune genes were expressed in EC and intersected with DEGs to obtain differentially expressed immune genes (DEIGs). Subsequently, DEIGs were used in univariate Cox regression analysis to identify significant prognosis-related immune genes, followed by Least absolute shrinkage and selection operator (LASSO) regression analysis to obtain independent prognostic genes. LASSO regression can improve the accuracy and interpretability of the model and also exclude the problem of collinearity between independent variables (Alhamzawi and Ali, 2018). Multivariate Cox regression analysis was conducted to obtain regression coefficients for independent prognostic factors. Finally, an immune risk score signature (IRSS) was established based on the multivariate Cox regression coefficient beta value, and the formula is as follows: an immune risk score signature (IRSS) = EXP*gene*1^∗^ β1 + EXP*gene*2 ^∗^β2 + EXP*gene*3^∗^β3 + … + EXP*gene*n^∗^βn, where EXP means expression level and β represents the regression coefficient from the multivariate Cox (Zeng et al., 2017).

By calculating the risk score for each sample of TCGA-ESCA, patients were divided into low- and high-risk groups using the median as the cut-off value. Furthermore, visualization of the Kaplan-Meier (KM) curve was utilized to compare OS between the two groups by the log-rank test. The area under the receiver operating characteristic (ROC) curve (AUC) was adopted for analyzing the prognostic predictive value of IRSS in patients with EC. The ROC curves are all referred to as the receiver operating characteristic curves, with sensitivity as the ordinate and 1-specificity as the abscissa (DeLong et al., 1988). The AUC is the probability value, which ranges from 0.5 to -1, used to evaluate the accuracy of the model prediction, and a larger area means higher accuracy. In the present study, the larger its value, the higher the degree to which the predicted overall survival agreed with the actual overall survival.

### Immune Risk Score Signature Combined With Clinicopathological Information

We screened for prognostic predictive factors, including clinical characteristics and established IRSS. Specifically, the univariate Cox proportional hazard model was employed to analyze the correlation between IRSS and OS, and the multivariate Cox regression analysis was used to evaluate whether the established IRSS could serve as an independent prognostic predictor. Further, to comprehensively assess patient survival, we constructed a nomogram integrating distinct clinicopathological information, including age, sex, disease type, stage, smoking, alcohol, BMI and IRSS, using the “rms” package. Additionally, the concordance index (C-index) was used to evaluate the predictive accuracy of the nomogram. Similarly, the decision curve analysis (DCA) of 2, 3, and 5 years was calculated to evaluate whether the synthetic nomogram established by us is suitable for clinical application. The x-axis represents the percentage of the threshold probability, and the y-axis represents the net income.

### Validation of IRSS

To assess the general applicability of the signature, Considering the anatomical and histological similarities, we selected the TCGA-HNSC (*n* = 500) to further validate the established model. The risk scores of each patient in the HNSC cohort were calculated and ranked using the formula of the IRSS established in TCGA-ESCA. HNSC samples that had been sorted by scores were divided into high- and low-risk groups according to the cut-off values obtained in the TCGA-ESCA cohort. KM curves were used for comparison of the survival differences between the two groups, and ROC curves were used to assess the accuracy of the signature prediction.

Similarly, the nomogram was used to comprehensively assess the survival probability of patients with HNSC, incorporating clinical information including age, gender, stage, smoking, alcohol, lymph nodes, and IRSS. Calibration curves (2-, 3-, and 5-year) were drawn to assess whether the predictive effect of the nomogram was accurate, and its 45° line represented the best predictive effect. In addition, the C-index was used to compare the accuracy of traditional TNM-stage, IRSS, and nomogram prediction. DCA was performed to evaluate the clinical value of the comprehensive nomogram for HNSC.

Further, to evaluate the prognostic value of the IRSS in gastric cancer (GC), which is an upper gastrointestinal tumor, we utilized the KM plotter online analysis website to validate the model^3^. This website contains multiple GEO databases of GC involving GSE62245, GSE14210, GSE15459, GSE22377, GSE29272, and GSE51105. We combined these databases to provide a prognostic assessment of overall survival based on genes in the IRSS in 631 patients with GC, respectively (Szasz et al., 2016).

### Statistical Analysis

Simple mathematical analysis and processing were completed by Excel software. Multivariate Cox regression analysis was performed by SPSS 20.0, with a probability of stepwise entry of 0.05 and removal of 0.1. Further data analysis and visualization are mainly accomplished by R (v3.6.1). Survival ROC curves were drawn by the “survival ROC” package in R. “Survival” packages were used to plot KM curves, C-index, as well as clinical univariate and multivariate regression analyses in R. Besides, visualization of DEGs, was accomplished by volcanoes drawn by the “ggplot2,” “Cairo,” and “ggrepel” packages. The *P*-value less than 0.05 was considered a statistically significant criterion.

## Results

### Differential Analysis

**FIGURE 1:**
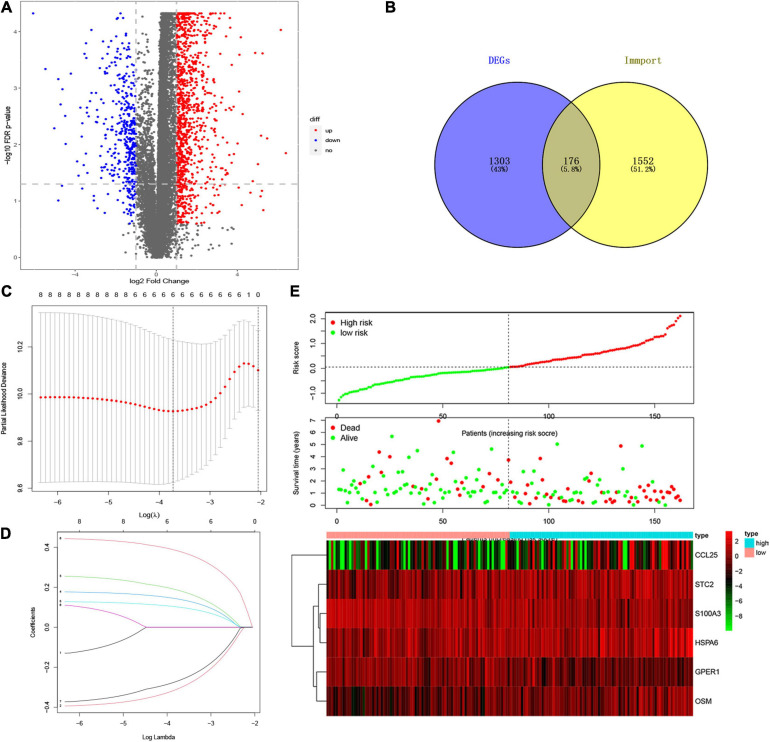
Establishment of IRSS Signature. **(A)** Volcano plot of 1479 differentially expressed genes. **(B)** Venn diagram of the intersection of DEGs and immune genes. **(C)** Ten-time cross-validation for tuning parameter selection in the LASSO model. **(D)** LASSO coefficient profiles. **(E)** The risk score, survival status, and heat map of six immune genes in patients with EC.

A total of 156 EC patients with prognostic and gene expression data and survival longer than 60 days were included in the training set, as well as 11 matched normal samples. To investigate a biomarker that can effectively predict the prognosis of EC, we established a risk score model based on immune genes to evaluate the outcomes of patients with EC. Specifically, we performed a differential analysis between normal and tumor groups and obtained genes significantly associated with EC. And a total of 1479 DEGs were identified, as shown in Supplementary Table 2, and visualized with volcano maps (Figure 1A).

### GO and KEGG Analysis

**FIGURE 2:**
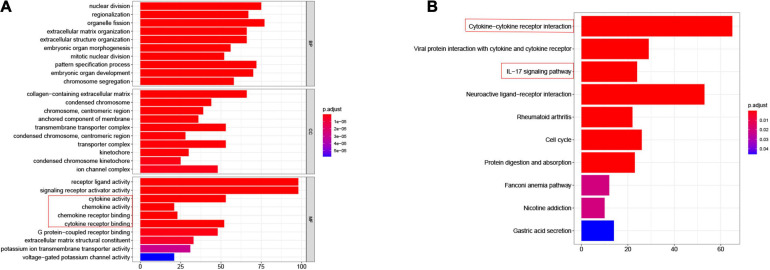
Differential genes expressed in normal and tumor groups of esophageal cancer are involved in immune-related pathways. **(A)** GO, Gene Ontology, immune related pathways marked by red boxes; **(B)** KEGG, Kyoto Encyclopedia of Genes and Genomes, immune related pathways marked by red boxes.

To explore the potential association between gene expression and immunity in normal and tumor groups in the TCGA-ESCA cohort, we performed GO and KEGG enrichment pathway analysis. The DEGs in normal and tumor groups were enriched in a variety of processes, most of which were in immune-related pathways. Specifically, Figure 2 shows the cytokine-cytokine receptor interaction and IL-17 signaling pathway in KEGG enrichment analysis and the molecular functional modules involved in chemokine activity, cytokine activity, chemokine and receptor binding in GO enrichment analysis. Therefore, these findings revealed that the occurrence of EC was related to the expression level of immune genes. Detailed enrichment analysis results are presented in the Supplementary Tables 3, 4.

### Construction and Prognostic Value of IRSS

To explore whether immune genes could be used as effective biomarkers to indicate the prognosis of EC, we selected immune-related genes from the DEGs for further analysis. The Venn diagram (Figure 1B and Supplementary Table 5) showed that 176 DEIGs were screened from the overlap of immune genes and DEGs. Subsequent univariate Cox regression analysis yielded 8 immune genes significantly associated with prognosis (Supplementary Table 6), followed by LASSO regression analysis. Combining the results of Figures 1C,D, it was considered that the model fit the best when the penalty coefficient was 6, and the corresponding six immune genes were selected into the model, which was *S100A3*, *STC2*, *HSPA6*, *CCL25*, *GPER1*, and *OSM* (Figure 1E and Supplementary Table 7). moreover, multivariate Cox regression analysis was performed on the six immune genes, which were still able to enter the equation as a prognostic predictor (Supplementary Table 8). Moreover, the corresponding regression coefficients were obtained, β*1*-β*6*, which were −0.400, 0.246, 0.177, 0.127, −0.349, and 0.442, respectively. According to the formula mentioned above, combined with the beta value of multivariate Cox regression, the IRSS was finally established:

IRSS=EXPS100A3*-0.400+EXPSTC2*0.246

+EXPHSPA6*0.177+EXPCCL25*0.127

+EXPGPER1*-0.349+EXPOSM*0.442

**FIGURE 3:**
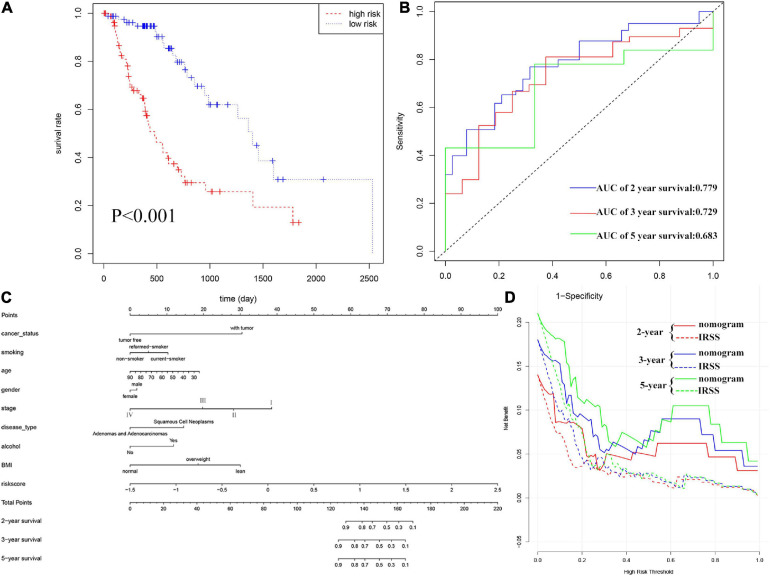
Evaluation of IRSS signatures and establishment and evaluation of nomograms. **(A)** Kaplan-Meier curves show that OS was significantly different between the high- and low-risk groups in TCGA-ESCA. **(B)** The signature is shown by the time-dependent ROC curve for predicting 2, 3, and 5-year survival. **(C)** 2-, 3-, and 5-year nomogram for predicting OS of EC. There are nine components in this nomogram: cancer status, smoking, age, gender, stage, histological type, alcohol, BMI, and IRSS. **(D)** Decision curve analysis for the evaluation of the net benefits of IRSS and nomogram at 2, 3, and 5 years.

Furthermore, according to the above formula, the risk score of each EC patient was directly calculated. And then, the samples were divided into high- and low-risk groups, which were grouped according to the median and interquartile range [M(IRQ) = 0.040 (−0.321, 0.588)]. The results of the KM curve showed that the prognosis of the high-risk group was worse than that of the low-risk groups (Figure 3A, log-rank *P* < 0.001; HR = 3.68, 95% CI = 2.14−6.35). ROC curves were employed to assess the accuracy of established models for predicting OS in patients with EC. As shown in Figure 3B, the AUC values of 2, 3, and 5 years were 0.779, 0.729, and 0.683, respectively, indicating the robustness and accuracy of the model in predicting patient prognosis.

### The Value of IRSS in Clinical Characteristics

To determine whether the established IRSS has prognostic significance, we further performed univariate and multivariate Cox regression analysis. Univariate Cox regression analysis showed that risk score, cancer status and stage were prognostic predictors in TGGA-ECSA, but not smoking, alcohol, age, sex, disease type, BMI, and radiation therapy. More importantly, the risk score was also observed to be the only independent predictor in multivariate Cox regression analysis (Table 1). The above results show that our established IRSS could serve as a robust and novel biomarker for predicting prognosis.

**TABLE 1:** Univariate/multivariate Cox regression analysis of clinicopathological features of EC associated with OS.

**Variables**		**Patient *N* (156)**	**Univariate analysis**		**Multivariate analysis**	
			**HR^*a*^ [95% CI]**	***P***	**HR [95% CI^*b*^]**	***P***
Age	<65	93	1			
	≥65	63	0.896 [0.536,1.497]	0.675		
BMI	Normal	8	1			
	Lean	57	2.322 [0.894,6.035]	0.084		
	Overweight	82	1.355 [0.734,2.503]	0.332		
Stage	Stage i	15	1		1	
	Stage ii	67	2.859 [0.661,12.360]	0.16		
	Stage iii	47	7.483 [1.699,32.966]	0.008		
	Stage iv	8	22.130 [4.493,109.001]	<0.0001*	1.286 [0.438,3.778]	0.647
Cancer status	Tumor free	60	1		1	
	With tumor	35	3.673 [1.394,9.676]	0.008*	2.164 [1.182,3.964]	0.516
Histological type	EA	79	1			
	ESCC	77	0.812 [0.482,1.366]	0.433		
Gender	Male	23	1			
	Female	133	2.236 [0.892,5.603]	0.381		
Smoking	Non-smoker	45	1			
	Current-smoker	32	1.677 [0.736,3.819]	0.218		
	Reformed-smoker	61	1.615 [0.785,3.319]	0.193		
Alcohol	No	44	1	0.181		
	Yes	110	0.703 [0.419,1.178]			
Radiation therapy	No	61	1			
	Yes	21	1.489	0.439		
IRSS		156	2.149 [1.666,2.772]	<0.0001*	2.319[1.615,3.330]	0.012*

^*a*^HR, hazard ratio. ^*b*^CI, confidence interval. *P < 0.05.

Nomograms, which simplify statistical prediction models to single numerical estimates of event probabilities tailored to individual patient profiles, are widely used for prognostic assessment of tumors (Iasonos et al., 2008). A variety of clinical features have prognostic value in clinical practice. Therefore, in order to accurately evaluate the prognosis of patients, we established a nomogram containing multiple clinicopathological characteristics as well as IRSS. As shown in Figure 3C, scores for each variable could be calculated and combined to comprehensively predict the prognosis of patients with EC.

The C-index of the established nomogram, risk signature, and TNM-stage was 0.881, 0.721, and 0.693 (Table 2), respectively. In summary, the predictive ability of our IRSS was stronger than that of the traditional TNM-stage, however, the predictive accuracy of the nomogram integrating multiple clinical information was the most robust. Consistent with this result, the DCA figure (Figure 3D) also proved that the nomogram combined with various clinical features has better clinical application value.

**TABLE 2:** The C-index values of the nomogram, TNM-stage, and IRSS.

**Cohorts**	**Variables**	**C-index (95%CI)**
EC	TNM-stage	0.693 (0.657,0.731)
	IRSS	0.721(0.688,0.754)
	nomogram	0.881 (0.822,0.940)
HNSC	TNM-stage	0.512 (0.493,0.531)
	IRSS	0.558 (0.534,0.582)
	nomogram	0.781 (0.759,0.803)

### Validation of Other Cancer Species

It is well known that HNSC and EC belong to malignant epithelial tumors of the upper gastrointestinal tract, which are characterized by early dissemination and poor prognosis (Sproll et al., 2018). To verify the general applicability of the IRSS, the data of the TCGA-HNSC cohort with similar tissue and anatomical characteristics was downloaded as the validation cohort. First, according to the IRSS formula obtained in the TCGA-ESCA cohort, the risk score was calculated for each patient in the HNSC cohort. Further, HNSC samples were divided into high and low-risk groups according to the median IRSS of the EC cohort.

**FIGURE 4:**
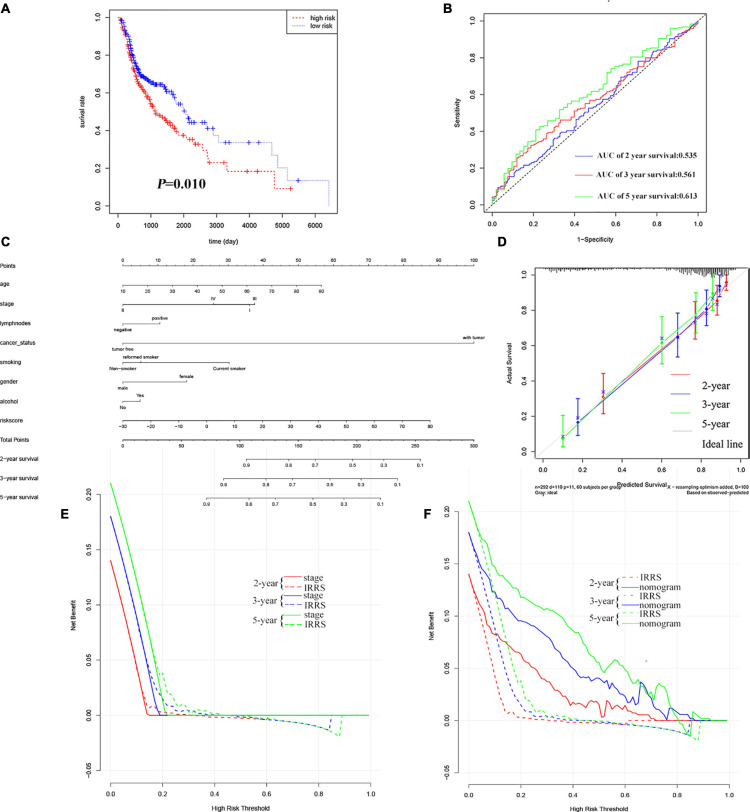
Validation of IRSS signature with TCGA-HNSC. **(A)** Kaplan-Meier curves show that OS in the low-risk was significantly higher than in the high-risk group. **(B)** Time-dependent ROC curve analysis of the IRSS at 2, 3, and 5 years. **(C)** 2-, 3-, and 5-year nomogram for predicting OS of HNSC. **(D)** The Calibration curve of the nomogram for predicting OS rate at 2, 3, and 5 years. **(E,F)** Decision curve analysis for the evaluation of the net benefits of TNM-stage, IRSS and nomogram at 2, 3, and 5 years.

The results of KM analysis (Figure 4A) could confirm that the low-risk group was associated with a better prognosis, while the high-risk group predicted a worse prognosis (*P* = 0.010; HR = 1.43, 95% CI = 1.09−1.88), which was consistent with the results of EC. The AUC of survival ROC curve shows that the model had good consistency in predicting OS and actual OS (Figure 4B, 0.535, 0.561, and 0.613 at 2, 3, and 5 years, respectively). Clinical characteristics and IRSS were used to establish a predictive nomogram for predicting the prognostic survival probability of HNSC patients at 2, 3, and 5 years (Figure 4C). The calibration curve results confirm that there is good consistency between the actual survival probability and the predicted probability (Figure 4D). The results of the C-index and DCA showed that IRSS had a better prognostic predictive ability for HNSC than traditional TNM-stage, but the comprehensive nomogram was the best (Figures 4E,F and Table 2).

To investigate the prognostic predictive value of the model in GC with upper gastrointestinal tumors, the IRSS model was further validated in gastric cancer. Combining multiple GEO databases of gastric cancer, KM-plot results indicated that 6 immune genes in IRSS were highly associated with the prognosis of GC, and each independent gene could likewise serve as a biomarker for predicting the outcome of GC (Supplementary Figure 1).

Taken together, the established IRSS had good applicability and could not only predict the prognosis of EC but also serve as a prognostic predictive biomarker for the upper gastrointestinal tumors.

## Discussion

Esophageal cancer remains one of the most lethal malignancies in the world with a poor prognosis (Ferlay et al., 2015). Over the past decades, the incidence of EC has increased markedly in many countries (Simard et al., 2012), ranking fourth among cancer deaths in China (Chen et al., 2016). Owing to the lack of early-onset symptoms, EC is usually diagnosed at an advanced stage. A variety of studies have found that the carcinogenic process of EC is closely correlated with the immune-inflammatory response (Lin et al., 2016). A major mechanism of inflammation-induced esophageal carcinogenesis is through structural activation of inflammatory signaling pathways (Abdel-Latif et al., 2009). EC cells are rich in tumor antigens, including tumor-associated antigens and neoantigens, and can initiate dendritic cell-mediated cytotoxic T lymphocytes early in tumorigenesis (Huang and Fu, 2019). Environmental exposure can trigger chronic esophageal inflammation, further promoting the activation of pro-inflammatory signaling pathways for survival and proliferation (Lin et al., 2016). The induction of these pathways leads to the activation of downstream gene transcription and enzyme activity, which play a key role in tumor growth and survival. Tumor immunotherapy is a promising new method for the treatment of EC, and different studies on EC immunotherapy have been carried out in recent years (Kelly, 2019). However, EC immunotherapy always results in mixed outcomes, partly because of the lack of reliable markers to predict treatment response (Huang and Fu, 2019). In the current study, we aim to establish immune-related biomarkers to effectively predict the outcome of EC.

To explore the relationship between EC and immune mechanisms, we selected the TCGA-ECSA database as a training set for analysis. To find the DEGs between the normal group and tumor group of EC to obtain gene annotation information, the differential analysis was carried out first. We then performed GO and KEGG enrichment analysis on the DEGs and the results showed that immune and tumor-related signaling pathways were significantly enriched. This is consistent with previous findings that immune inflammation induction is an important mechanism of esophageal carcinogenesis (Abdel-Latif et al., 2009). Therefore, we will further explore the potential role of immunological biomarkers in tumor prognosis.

Next, we select the immune genes among the DEGs and obtain 6 independent immune genes related to prognosis according to the Cox proportional hazard model and lasso regression analysis. These six immune genes were integrated to construct an IRRS that can effectively predict prognosis. Among these genes, *S100A3* belongs to the S100 family and is considered to be associated with a good prognosis of ovarian cancer (Bai et al., 2018), which is similar to our results. However, in gastric cancer, the high expression of *S100A3* is closely in relation to the poor survival of patients (Wang et al., 2019). *STC2* (stanniocalcin 2), whose expression in ESCA was higher than that in corresponding normal tissues, was significantly associated with lymph node metastasis, lymphatic invasion and distant metastasis (Kita et al., 2011; Kashyap et al., 2012), and has been reported as a prognostic glycolysis-related gene in HNSCC (Ferreira do Carmo et al., 2020; Liu and Yin, 2020). *HSPA6*, a heat shock protein, was considered to be associated with the recurrence of human hepatocellular carcinoma in the study of Yang et al. (2015). Zhang et al. (2016) have reported that *CCL25* (C-C chemokine receptor ligand 25) may promote the migration and invasion of cancer cells by affecting several Epithelial-mesenchymal transition (EMT) markers and providing the chemotactic ability for hepatocytes and breast cancer cells through the CCL25/CCR9 signaling pathway. *GPER1* (G-protein-coupled estrogen receptor 1) is recognized as a key regulator of immune-mediated events in breast, pancreatic, prostate and hepatocellular carcinomas, as well as melanoma (Notas et al., 2020). *OSM* has been reported to have diagnostic, prognostic, and therapeutic capabilities in a variety of diseases (Verstockt et al., 2019). For example, Tawara et al. (2018) argue that early therapeutic inhibition of *OSM* in breast cancer patients is thought to prevent breast cancer metastasis.

In this study, the results of KM analysis showed that IRSS was an effective biomarker for predicting the prognosis of EC. Significant differences in OS were observed between the high- and the low-risk group, implying that the high-risk group was associated with adverse outcomes. Furthermore, the survival ROC results showed that the predictive effect of our model on prognosis was in good agreement with the actual results. Additionally, survival analysis with multiple clinicopathological information, age, sex, tissue type, stage, smoking, alcohol consumption, BMI and radiation therapy as covariates, demonstrated that the established model remained a robust independent prognostic predictor. In order to evaluate the prognosis comprehensively, we combined a variety of clinical information and established a nomogram to score the survival probability of each patient. The results of the DCA and C-index showed that the prediction accuracy of IRSS was higher than that of the traditional TNM-stage, however, the nomogram integrating multiple clinical information could predict the prognosis of EC patients more accurately.

Head and neck cancers, mainly including two histological subtypes of head and neck adenocarcinoma (HNA) and head and neck squamous cell carcinoma (HNSCC) (Andreasen et al., 2019). HNSCC is not only close to EC in histological classification and anatomical location but also has many similar carcinogenic factors. Chronic inflammation and microbial dysbiosis, including HPV infection (de Villiers et al., 2004), Porphyromonas gingivalis infection, and their synergistic effects with alcohol and tobacco (Olsen and Yilmaz, 2019), are closely associated with the occurrence of oral and digestive cancers, including (larynx, throat, lip, mouth, and salivary glands) and ESCA. Additionally, overexpression of the Dek oncogene in SCC (squamous cell carcinoma)-derived human keratinocytes can promote the development of ESCA and HNSC *in vivo* (Matrka et al., 2018). Considering the similarity of histological type, anatomical location and pathogenic factors, we utilized TCGA-HNSC as a validation cohort to evaluate the prognostic predictive value of the established model for these two tumors. Interestingly, the IRSS we constructed can not only be used as a prognostic biomarker for EC but also be used to predict the outcome of HNSC, which shows that the signature has wide robustness and applicability. Moreover, this may provide a new idea for the treatment of EC.

Currently, potential biomarkers for predicting prognosis have been widely used in EC and other cancers (Li et al., 2019; Lu et al., 2020). For instance, Qu et al. (2020). comprehensively analyzed the tumor microenvironment of cutaneous melanoma by using ESTIMATE and identified genes associated with the tumor microenvironment as biomarkers and their correlation with the immune system (Pan et al., 2019). As we prepared this paper, a study on the immune risk model of EC has been established and published (Guo et al., 2020). However, compared with this literature, our differential analysis screening criteria are more stringent. The number of prognosis-related immune genes obtained was different due to different screening criteria, but the overlapping two genes, *OSM* and *HSPA6*, confirmed the reliability of our established model. In addition, the clinicopathological factors considered in our nomogram, including smoking, alcohol consumption, disease type, BMI, and tumor status, enable a comprehensive assessment of the prognostic survival probability of patients with esophageal cancer. Besides, the dataset TCGA-HNSC was used as a validation set to confirm the applicability, robustness, and prognostic value of the model in upper gastrointestinal malignancies. Therefore, compared with the former, our study has further research progress and clinical significance.

In this study, the potential relationship between immunity and EC was explored. Based on six immune genes, a novel and robust biomarker for predicting the prognosis of EC and HNSC was established and validated. The signature proved to be an independent prognostic biomarker, which may provide a potential therapeutic target for the clinical treatment of upper gastrointestinal cancers such as EC, GC and HNSC, as well as ideas for the study of their correlation.

## Data Availability Statement

The original contributions presented in the study are included in the article/Supplementary Material, further inquiries can be directed to the corresponding author/s.

## Author Contributions

CZ and QX conceived the study, performed the data analysis, and wrote the manuscript. BG downloaded the gene expression data of esophageal cancer. XL, HJ, and SW critically revised the manuscript for research content and administrative support. All authors read and approved the final manuscript.

## Conflict of Interest

The authors declare that the research was conducted in the absence of any commercial or financial relationships that could be construed as a potential conflict of interest.

## Footnotes

1 https://tcga.xenahubs.net

2 https://immport.niaid.nih.gov/

3 http://kmplot.com/analysis/
