# Supplementary figures and images for: Esophageal Cancer Associated Immune Genes as Biomarkers for Predicting Outcome in Upper Gastrointestinal Tumors
Source: Front Genet. 2021 Jul 19;12:707299. doi: 10.3389/fgene.2021.707299 (PMC8327216; doi:10.3389/fgene.2021.707299)

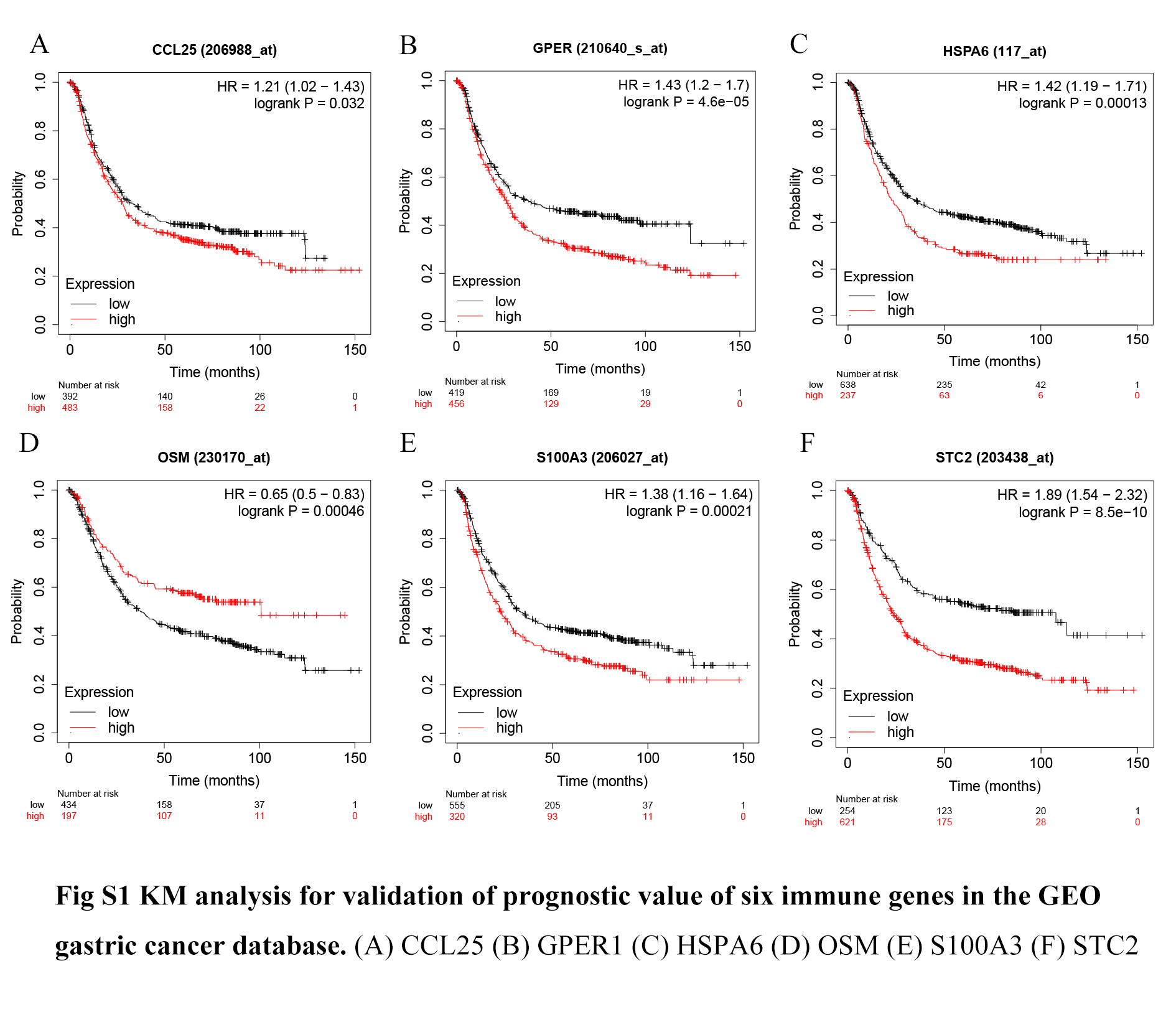

Supplement: Supplementary file 2 [file Image_1.TIF]
